# Pharmacokinetic/pharmacodynamic profiles of baicalin against *Mycoplasma gallisepticum* in an in vivo infection model

**DOI:** 10.1016/j.psj.2021.101437

**Published:** 2021-08-20

**Authors:** Jiaxin Bao, Zhiyong Wu, Muhammad Ishfaq, Jian Wang, Yusong Miao, Dong Niu, Rui Li, Jichang Li, Chunli Chen

**Affiliations:** ⁎ College of Veterinary Medicine, Northeast Agricultural University, Harbin 150030, P. R. China; † Heilongjiang Key Laboratory for Animal Disease Control and Pharmaceutical Development, Harbin 150030, P. R. China.; ‡ College of Computer Science, Huanggang Normal University, Huanggang, 438000, P. R. China

**Keywords:** *Mycoplasma gallisepticum*, baicalin, PK/PD, chicken

## Abstract

*Mycoplasma gallisepticum* (***M. gallisepticum***), a devastating avian pathogen that commonly causes chronic respiratory disease in chicken, is responsible for tremendous economic losses to the poultry industry. Baicalin is the main constituent of *Scutellaria baicalensis* that shows potential therapeutic effects against *M. gallisepticum*. However, the pharmacokinetic/pharmacodynamics (**PK/PD**) profiles of baicalin against *M. gallisepticum* are not well understood. The main objective of the present study was to determine the relationship between the PK/PD index and efficacy of baicalin in the *M. gallisepticum* infection model in chickens. The experiments were carried out on 10-day-old chickens that were challenged with *M. gallisepticum* in the bilateral air sacs. While, baicalin was orally administrated once in a day for 3 consecutive days, started from d 3 postinfection. Ultra-performance liquid chromatography (**UPLC**) was used to evaluate the PK parameters of baicalin at doses of 200, 400, and 600 mg/kg in *M. gallisepticum*-infected chickens. Real-time PCR (**RT-PCR**) was used for the quantitative detection of *M. gallisepticum* in lungs. The PK and PD data were fitted to WinNonlin software to evaluate the PK/PD profiles of baicalin against *M. gallisepticum*. The minimum inhibitory concentration (**MIC**) of baicalin against *M. gallisepticum* strain R_low_ was 31.25 µg/mL. The in vivo data suggested that baicalin concentration in the lung tissues was higher than plasma (1.21–1.73 times higher). The ratios of AUC_24h_/MIC of baicalin against bacteriostatic, bactericidal, and eradication were 0.62, 1.33, and 1.49 h, respectively. In conclusion, these results provided potential reference for future clinical dose selection of baicalin and evaluation of susceptibility breakpoints.

## INTRODUCTION

*Mycoplasma gallisepticum* (***M. gallisepticum***) is one of the serious pathogens that harm the global poultry industry. *M. gallisepticum* is considered one of the primary causative agents of chronic respiratory disease in chicken (Stanley et al., 2001; Beaudet et al., 2017). Its primary signs include shedding of tears, sneezing, increased nasal discharge, and other respiratory symptoms. The transmission route of *M. gallisepticum* includes horizontal route via aerosol and vertical route via eggs. It has been previously demonstrated that *M. gallisepticum* infection suppressed the host immune response and increased the chances for secondary bacterial infections (Zhiyong et al., 2019; Wu et al., 2020; Wang et al., 2021b). It causes considerable economic losses in poultry industry in terms of reduced egg production, feed conversion rates and high mortality rates (Zhang et al., 2021). Currently vaccination and antibiotics are used to control *M. gallisepticum* infection (Kanci Condello et al., 2020). However, it is difficult to eradicate the rapid spread of *M. gallisepticum* infection. Therefore, there is an urgent need to find better therapeutic agents.

Baicalin, a flavonoid compound possesses potential therapeutic properties, one of the main constituents of *Scutellaria baicalensis*. Previous studies demonstrated that baicalin has a variety of biological activities, such as antimicrobial, anti-inflammatory, antioxidant, antihepatotoxic, and antitumor properties (de Oliveira et al., 2015; Gong et al., 2017; Fu et al., 2018). Baicalin has the ability to interact with various signaling pathways. Our previous study explained the preventive effects of baicalin against *M. gallisepticum*-induced inflammation, oxidative stress, and apoptosis by activating the Nrf2 signaling pathway and NF-κB signaling pathway (Ishfaq et al., 2019), and baicalin could protect the immune organs from *M. gallisepticum* infection-mediated structural and functional damage (Zhang et al., 2020; Hu et al., 2021; Ishfaq et al., 2021). However, the lower bioavailability of baicalin limits its clinical uses. Numerous studies have been conducted on the pharmacokinetic profile of baicalin to clarify its in vivo properties (Wei et al., 2016), but it is difficult to obtain the connection between the efficacy and exposure concentration in vivo. These questions may be addressed through simulations from pharmacokinetic and pharmacodynamic models.

Pharmacokinetic-pharmacodynamic (**PK/PD**) modeling is a better method to understand the underlying mechanisms of drug action and the relationship on the physiologic system compared with single analytical method. It is well-known that PK/PD models, which have been used extensively in clinical trial design and the selection of dose regimens, provides useful information in optimizing the clinical dosage, improving the therapeutic efficacy and prohibiting resistance emergence (Nielsen and Friberg, 2013). In this study, we evaluated the PK/PD profiles of baicalin using *M. gallisepticum* infection model in chickens. Furthermore, we determined the better PK/PD index that correlated with the efficacy of baicalin. It was expected that our investigation would illustrate the relationship between dosing regimens and efficacy of baicalin, and assist further clinical development of optimal dosing strategies.

## MATERIALS AND METHODS

### Ethical Statement

The present study was conducted under the approval of the Laboratory Animal Ethics Committee of Northeast Agricultural University (Heilongjiang province, China) by Laboratory animal-Guideline for ethical review of animal welfare (GB/T 35892-2018, National Standards of the People's Republic of China).

### Mycoplasma Strains and Chemicals

The *M. gallisepticum* strain R_low_ was obtained from Harbin Institute of Veterinary Medicine, Chinese Academy of Agricultural Sciences. The culture conditions for growing *M. gallisepticum* were kept the same as mentioned in our previous study (Lu et al., 2017). Briefly, *M. gallisepticum* was grown at 37°C in modified Hayflicks medium containing 0.05% Penicillins, 0.1% Nicotinamide adenine dinucleotide (**NAD**), 10% freshly prepared yeast extract, 20% fetal bovine serum and 0.05% thallium acetate. At mid-exponential phase of *M. gallisepticum*, a color change was observed from phenol red to orange. Its concentration was adjusted at a density of 5 × 10^9^ color change unit per milliliter (**CCU/mL**) and kept at °C for use within 2 wk.

The reference standards of baicalin with a purity of above 98%, was purchased from the National Institute for the Food and Drug Control (Beijing, China). Methanol and Acetonitrile was of CHROMASOLV gradient grade for HPLC and obtained from Merck. LC/MS-grade Concentrated phosphoric acid and triethylamine were supplied by VWR.

### Susceptibility Determination

The in vitro susceptibility of baicalin against *M. gallisepticum* R_low_ was determined by the Microdilution method: 10 μL samples of *M. gallisepticum* culture (5 × 10^9^ CCU/mL) were inoculated onto 96-well plates containing 2-fold serial dilutions of baicalin. Growth control (*M. gallisepticum* inoculum without baicalin) and sterility control (blank medium) were also included in the MIC determination. The MIC was determined as the minimal concentration of the antibacterial agent that resulted in no growth on the 96-well plates after 7 d.

### Animals and Inoculation

Three hundred 1-day-old Hyline variety of White Leghorn chickens were bought from the Harbin Yinong farming Co., Ltd. (Harbin, China). Chickens were housed in a positive-pressure fiberglass isolator, and provided with antibacterial-free balanced feed and fresh drinking water ad libitum. The antibacterial-free chicken feed was purchased from Lenong Feed Co., Ltd. (Harbin, Heilongjiang, China). The chickens were randomly divided into 4 groups: 1) A group of 50 chickens were used as negative control group; 2) 20 chickens were used as a *M. gallisepticum*-infected group and challenged with *M. gallisepticum* strain R_low_ (1 × 10^9^ CCU/mL) in the bilateral air sacs of the thoracic region on d 10. The *M. gallisepticum*-infected group and negative control group were administrated by oral gavage with 0.85% NaCl started on d 13, once daily for 3 d; (3) A group of 180 chickens were used to study the pharmacokinetics of baicalin in infected chickens (infection route is same as described above, and the baicalin treatment started on day 13 in 3 different doses for 3 d consecutively, and 60 chickens were orally administrated for each dose once in a day). 4) A group of 50 chickens were used to study the pharmacodynamics of baicalin in infected chickens (the baicalin treatment was administered on d 13 in 8 different doses for 3 consecutive days, and 6 chickens were orally administrated once in a day for each dose separately). At the 16th d, all chickens were humanely sacrificed to avoid pain and suffering of chickens. Plasma and lung samples in each group were collected for further analysis.

### *M. Gallisepticum* Quantitation by Real-Time qRT-PCR

Quantitative real-time polymerase chain reaction (**qRT-PCR**) was used to identify DNA copies of *M. gallisepticum* in different samples using a Roche LightCycler instrument (Shanghai, China) with *M. gallisepticum*-specific *mgc2* gene. The primers are: *mgc2*-F: 5’-TTGGGTTTAGGGATTGGGATT; *mgc2*-R: 5’-CCAAGGGATTCAACCATCTT, as described in a previous study (Raviv and Kleven, 2009). Lungs were collected, homogenized in 2 mL PBS and centrifuged at 500 rpm for 5 min. An aliquot of 0.5 mL supernatant was used for DNA extraction with a bacterial DNA kit (Omega Bio-tek, Inc., Norcross, GA). The in vitro standard DNA curve was plotted by the numbers of *M. gallisepticum* derived from the culture method and cycle threshold (**Ct**) values obtained using qRT-PCR results as previously described (Wang et al., 2020).

### Efficacy of Baicalin Against *M. Gallisepticum* in Chicken Infection Model

To evaluate the efficacy of baicalin at 24 h post 3-d infection, the infected chickens were administrated baicalin orally by gavage with either 0.85% NaCl (controls) or baicalin at 100, 150, 200, 250, 300, 400, 500, or 600 mg/kg once daily for 3 consecutive days (6 chickens/dose). After 24 h of the last drug administration, the amounts of DNA copies of *M. gallisepticum* in each chicken were calculated using the method as described above.

### Baicalin Pharmacokinetics in In Vivo Infection Model

Groups of infected chickens were orally administrated baicalin by gavage at a dose of 200 mg/kg, 400 mg/kg, 600 mg/kg once daily for 3 d, and they were euthanized at 0.17, 0.25, 0.5, 0.75, 1, 2, 3, 6, 8, 12, and 24 h after the first oral gavage of baicalin. Blood and lung tissues were collected from 5 chickens at each sampling time point per treatment group. Blood samples were centrifuged at 3,000 × *g* for 10 min at °C, and then plasma was collected. The samples of plasma and lung tissues were stored at −20 °C until analyzed by UPLC within 2 wk.

Baicalin concentrations in plasma and lung tissues were determined by Ultra-Performance Liquid Chromatography (**UPLC**) (Waters Technologies, Shanghai, China). The UPLC was equipped with a Waters BEH-C18 column (2.1 mm × 50 mm, 1.7 μm) using a mobile phase of triethylamine phosphate (pH 2.4): Methanol (47:53, v/v) and a flow rate of 0.8 mL/min. Injection volume was 6 µL. The calibration curve was established with seven baicalin concentrations in plasma and lung tissues respectively.

Plasma and lung tissues were treated with three times the volume of methanol-acetonitrile mixed solution, vortexed for 2 min and incubated at 45°C in a water bath for 10 min to precipitate the proteins. Then, the samples were centrifuged at 12,000 *g* for 10 min. The supernatant was collected and the residue was extracted again. The extracts were combined and evaporated to dryness under a gentle stream of nitrogen at 40°C. The residue was dissolved in 200-µL mobile phase, and filtered through a 0.22 µm syringe filter prior to UPLC analysis. The recovery and precision were calculated by analysis of spiked samples at 3 concentration levels (5 replicates of each concentration).

### Pharmacokinetics and Pharmacodynamics Analysis

The PK profiles of baicalin were analyzed by the noncompartmental model with uniform weighting using the WinNonlin software (version 6.1; Pharsight, CA). The surrogate index of antibacterial activity, AUC_24h_/MIC and C_max_/MIC were calculated using in vitro MIC value and PK parameters obtained from 3 doses of baicalin. The bacterial load for each animal was calculated based on Ct values and the in vitro standard DNA curve. The efficacy of baicalin was evaluated by the reduction of *M. gallisepticum* load after treatment compared with the initial bacterial count before treatment. The PK/PD relationship of baicalin against *M. gallisepticum* was described by WinNonlin software (version 6.1; Pharsight). Linear, E_max_ and Sigmoid E_max_ models were chosen as candidate models. A dose-response relationship for baicalin was detected by multiple contrast tests, and then we choose the Sigmoid E_max_ model as the best-fit model in terms with the following equation:

$$E=E_{0}+\frac{E_{\max}\times C_{e}^{N}}{EC_{{}_{50}}^{N}+C_{e}^{N}}$$

where E is the change in Log_10_ CCU/mL for different dosage regimens, E_0_ is the change in Log_10_ CCU/mL in the control sample (absence of baicalin), E_max_ is the difference in effect between the greatest amount of growth (as seen for the growth control, E_0_) and the greatest amount of kill, Ce is the AUC_24h_/MIC in the effect compartment, EC_50_ is the AUC_24h_/MIC value producing a 50% reduction in bacterial counts, and N is the Hill coefficient that describes the steepness of the curve.

## RESULTS

### Susceptibility Testing

The MIC of baicalin against the studied strain was 31.25 μg/mL.

### *M. Gallisepticum* Infection Model

The model was successfully developed after 3 d of infection. These signs were observed including coughing, sneezing, nasal exudation and tracheitis in infected birds. Necropsy examination revealed cloudiness of air sacs and inflammation of the lungs. On the 13th day, the morbidity and mortality rates were 96 and 20%, respectively. While, the clinical signs of disease or *M. gallisepticum*-induced antibody were not observed in the negative control group.

### UPLC Analyses

**Figure 1:**
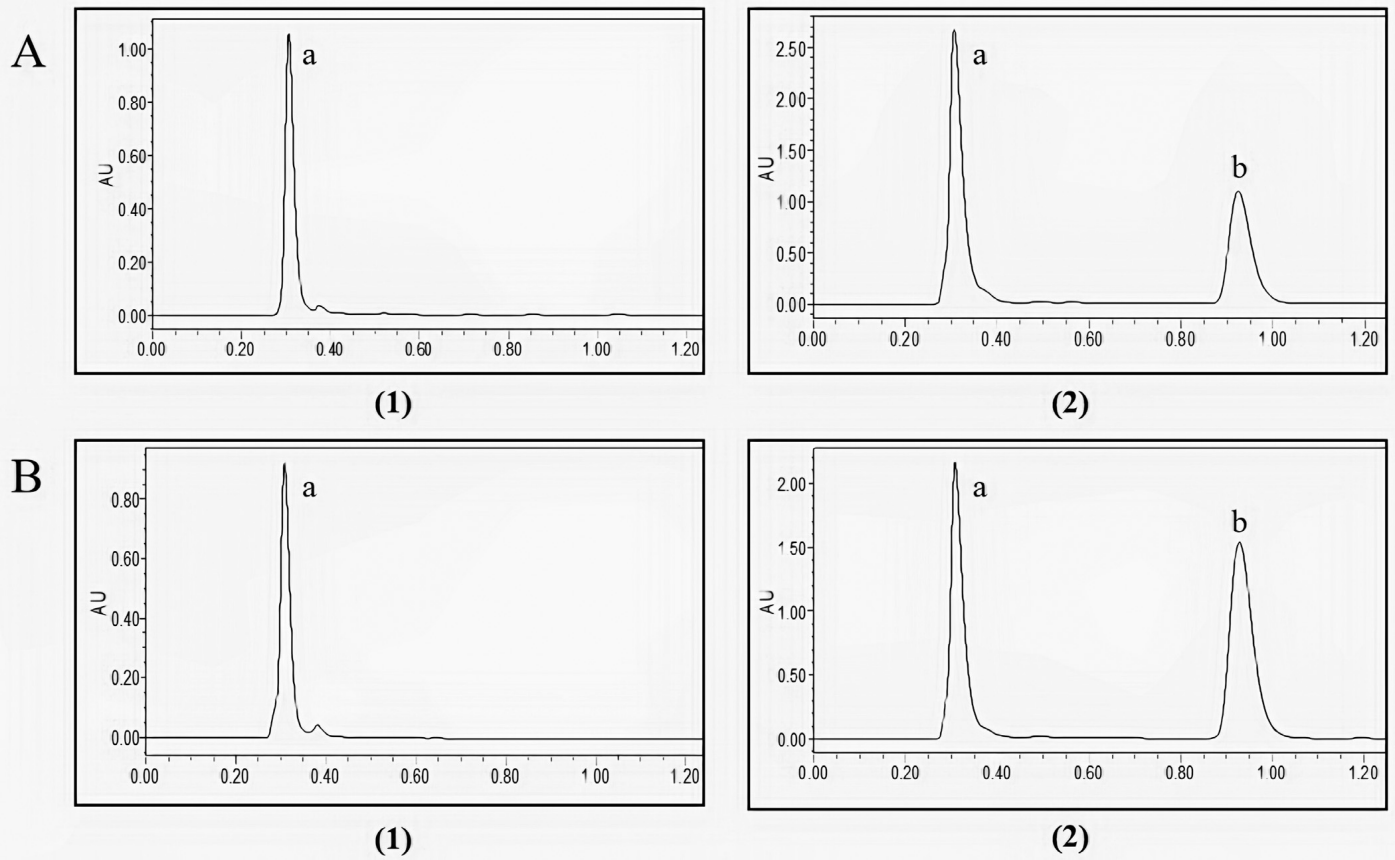
Typical chromatograms obtained in samples: (A) Plasma; (B) lung; (1) blank samples; (2) samples with addition of standard reference of baicalin; (a) the peak of endogenous substance; (b) the peak of baicalin.

The selectivity of the method was obtained by comparing the control samples before administration with the samples after addition of the baicalin standard. Figure 1 shows that UPLC method had a good selectivity. The linear regression equation is showed in Table 1, and baicalin in the plasma and lung homogenates all had good linearity within the concentration range. The intra and interday precision and accuracy were showed in Table 2. The precision of baicalin calculated as the relative standard deviation (**RSD**) at various concentrations. It has been noted that RSD was lower than 14% for intra- and interday experiment. The results showed that the precision was acceptable. The recovery of baicalin was higher than 85%, the LOD was 0.2 μg/mL and the LOQ was 0.05 μg/mL in the samples. These results suggested that excellent sensitivity and reproducibility were achieved under the above condition.

**Table 1:** The standard curve of baicalin in plasma and lungs.

Samples	Regression equations	Correlation (r)	Linearity range (μg/mL)
Plasma	Y = 37930X - 3125.2	0.9993	0.049–25.00
Lung	Y = 27173X - 49727	0.9997	0.024–25.00

**Table 2:** Precision and recovery of the baicalin in plasma and tissues in *M. gallisepticum* infection model.

			RSD (%)	
Samples	Concentration (μg/mL)	Recovery (%)	Intraday (n = 5)	Interday (n = 3)
Plasma	1.5	95.47 ± 2.12	2.22	7.14
	12.5	89.41 ± 1.82	2.03	6.13
	50	97.96 ± 2.60	2.65	7.30
Lung	2.5	98.84 ± 0.95	1.13	11.70
	25	85.01 ± 2.08	2.80	9.74
	100	88.83 ± 2.29	2.64	8.47

### PK Profiles of Baicalin in Infection Model

**Figure 2:**
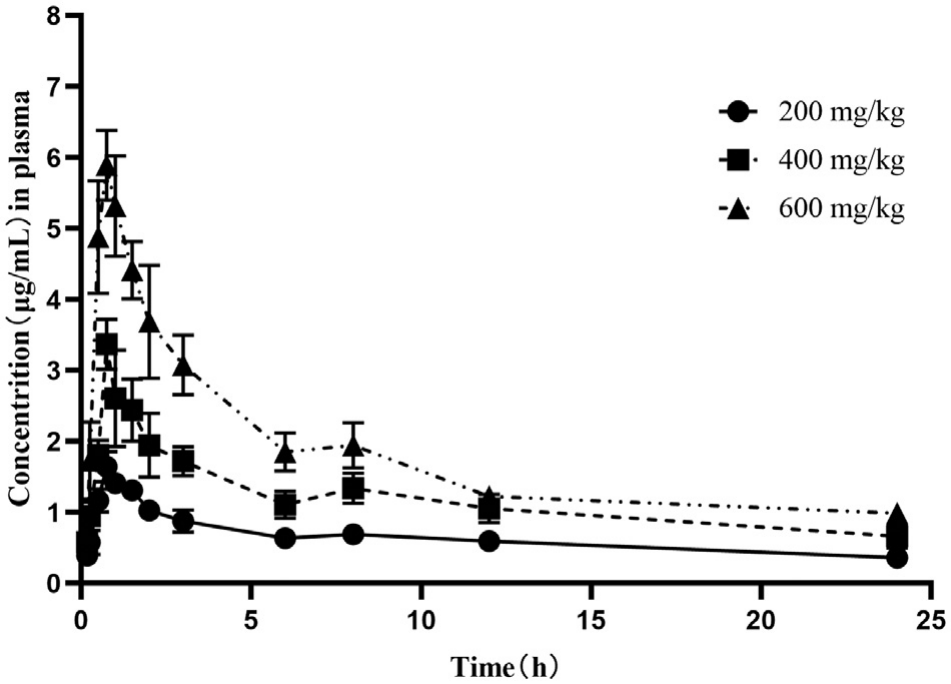
The time-concentration curves of baicalin in plasma after three oral administrations doses of 200, 400, and 600 mg/kg in *M. gallisepticum* infection model (n = 5/time point).

**Figure 3:**
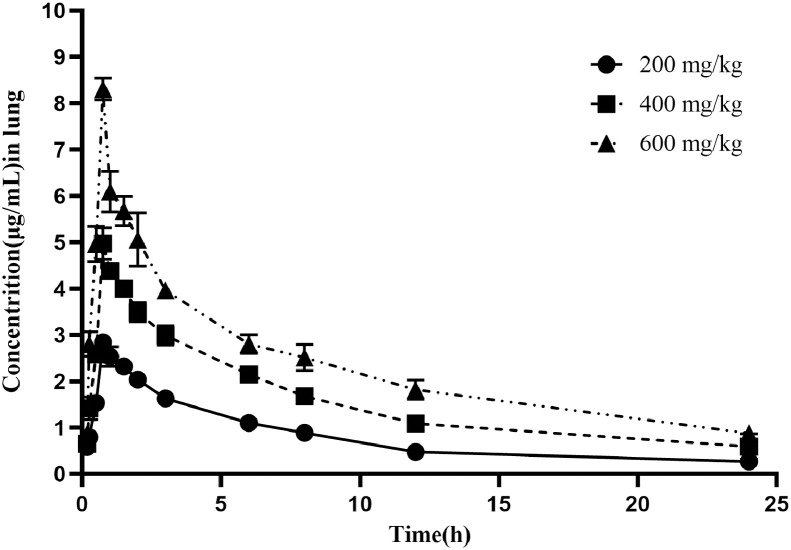
The time-concentration curves of baicalin in lung after three oral administrations doses of 200, 400, and 600 mg/kg in *M. gallisepticum* infection model (n = 5/time point).

**Figure 4:**
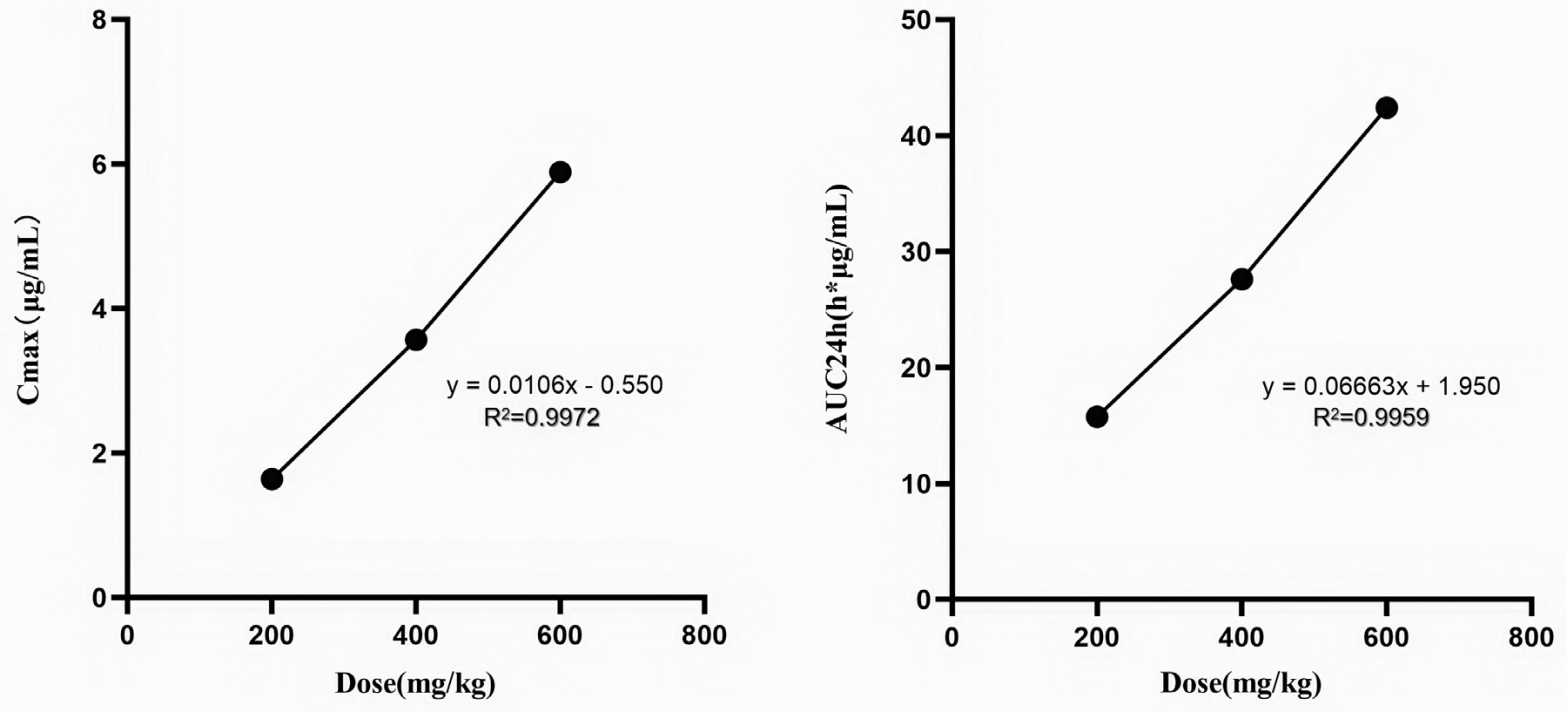
Linear regression plots between administered dose and C_max_ values, and between administered dose and AUC_24h_ values.

The time-concentration curves of baicalin in plasma and lung tissues after 3 oral administrations at a dose of 200, 400, and 600 mg/kg are shown in Figure 2, Figure 3, respectively. The main PK parameters obtained from plasma and lung tissues are presented in Tables 3 and 4, respectively. The T_max_ was 0.75 h in plasma and lung tissues for 3 different doses. A second peak was observed for all the doses administered at 7 to 9 h in plasma. The mean half-life (T_1/2_) was 14.45 ± 3.44 h in plasma and 8.75 ± 1.93 h in lung tissues. There were significant differences in T_1/2_ between lung and plasma. Besides, the PK parameters were dose-dependent, a significant correlation between dose and AUC_24h_ or C_max_ was observed (R^2^ = 0.996 and 0.997 for dose-to-AUC_24h_ and dose-to-C_max_ ratios, respectively; Figures 4). The AUC_24h_ values at different doses were calculated according to the linear relationship within the dose range from 100 to 600 mg/kg.

**Table 3:** Pharmacokinetic parameters of baicalin in plasma tissues following oral administrations of various doses in *M. gallisepticum*-infected chickens.

Dose (mg/kg)	T_max_ (h)	C_max_ (μg/mL)	AUC_24h_ (h × μg/mL)	T_1/2_ (h)	V/F (mL)	CL/F (L/h/kg)	MRT (h)
200	0.75	1.64	16.19	17.80	208.17	8.10	9.55
400	0.75	3.37	28.24	14.63	207.48	9.83	9.30
600	0.75	5.89	58.81	10.93	162.99	10.34	8.63

**Table 4:** Pharmacokinetic parameters of baicalin in lung tissues following oral administrations of various doses in *M. gallisepticum*-infected chickens.

Dose (mg/kg)	T_max_ (h)	C_max_ (μg/mL)	AUC_24h_ (h × μg/mL)	T_1/2_ (h)	V/F (mL)	CL/F (L/h/kg)	MRT (h)
200	0.75	2.84	19.02	6.86	91.77	9.26	7.24
400	0.75	4.84	36.99	8.67	112.72	9.02	7.86
600	0.75	8.31	55.09	10.72	135.11	8.73	8.06

### PD of Baicalin in Infected Chicken model

**Figure 5:**
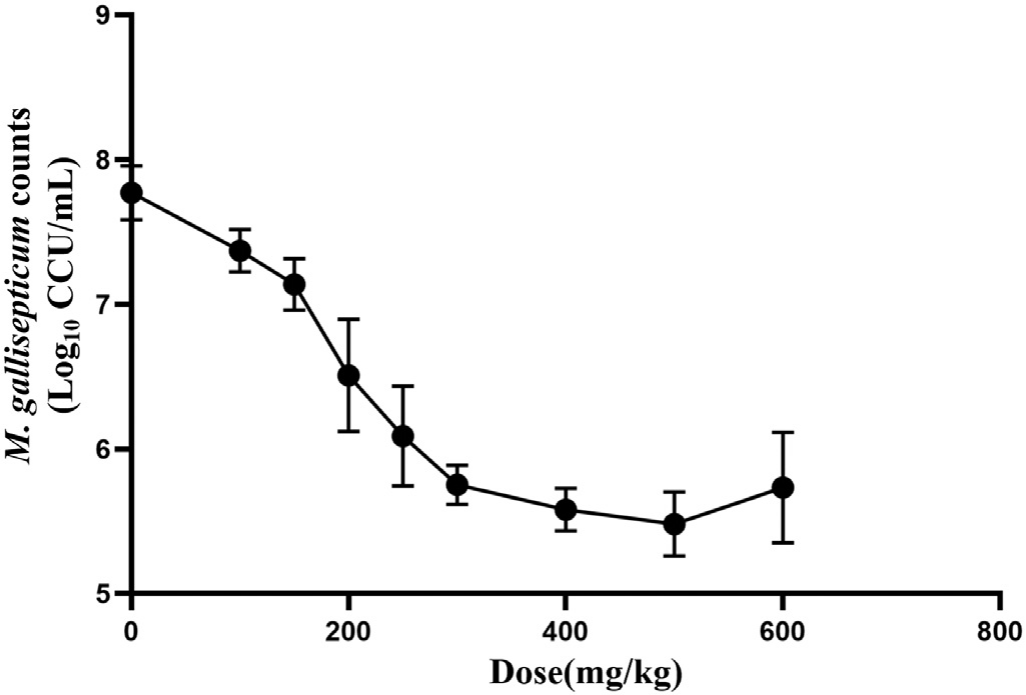
Calculated in vivo *M. gallisepticum* counts after baicalin treatment (n = 6/dose).

The effects of baicalin against *M. gallisepticum* in lung tissues with different regimens are shown in Figures 5. The data indicated that as the baicalin concentration increased from 100 to 600 mg/kg results in decrease in the bacterial load, implying that antimycoplasmal activity of baicalin was increased.

### PK and PD Analysis

**Figure 6:**
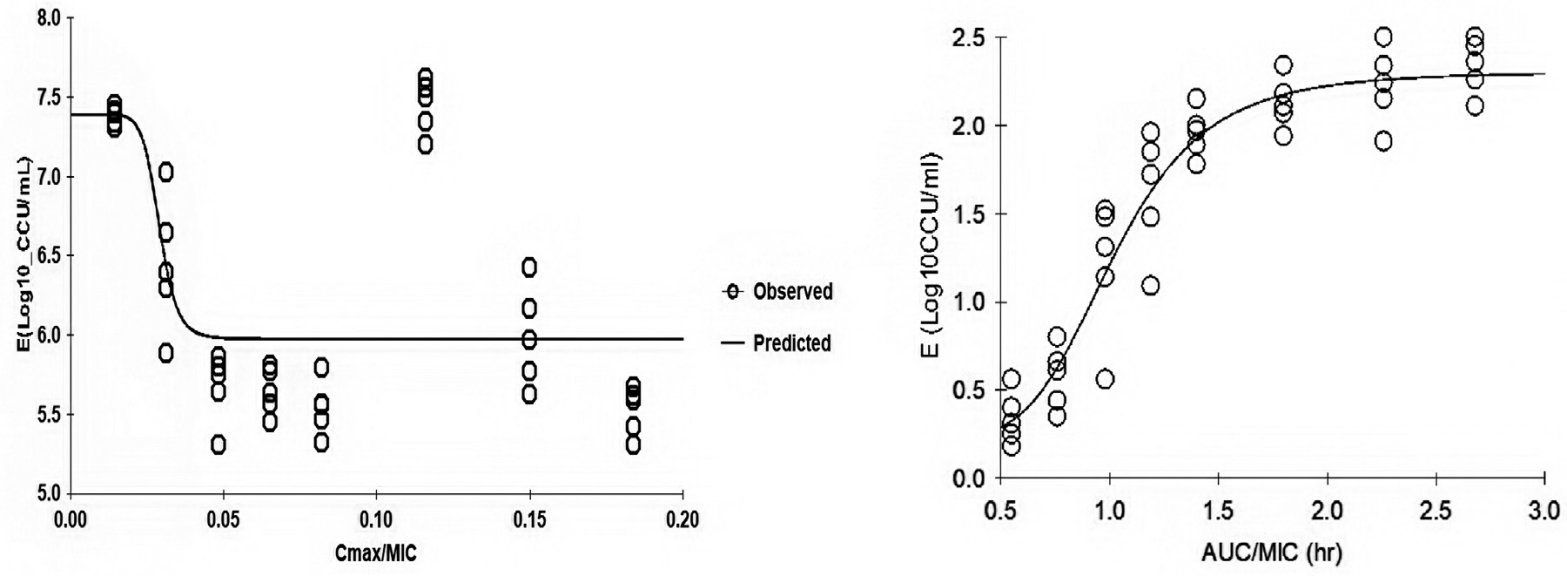
Sigmoid E_max_ relationships between antimycoplasmal effect (E, Log_10_ CCU/mL) and in vivo C_max_/MIC and AUC_24h_/MIC ratio against *M. gallisepticum* in the lung tissues of chickens.

The PK/PD indices AUC_24h_/MIC and C_max_/MIC were integrated using the PK parameters, dose proportionality, and MIC data. The effect (**E**) was calculated as the reduction of *M. gallisepticum* using the unit of Log_10_ CCU/mL. The relationship between the ratio of AUC_24h_/MIC and the ratio of C_max_/MIC with efficacy was described using the Sigmoid E_max_ model (Figure 6). The efficacy of baicalin correlated best with the AUC_24h_/MIC ratio with an R^2^ value of 0.959, followed by the C_max_/MIC (R^2^ = 0.898). The model parameters of Hill coefficient, N, E_max_ and AUC _24h_/MIC are presented in Table 5. The values of the AUC_24h_/MIC ratio required for bacteriostatic activity (E = 0), bactericidal activity (E = −2), and bacterial elimination (E = −3) were 0.62, 1.33, and 1.49 h, respectively.

**Table 5:** PK/PD analysis of baicalin in *M. gallisepticum* infection model.

Parameters	Value
E_max_ (Log_10_ CCU/mL)	2.30
E_0_ (Log_10_ CCU/mL)	1.03
EC_50_ (h)	1.03
AUC_24h_/MIC for 0 Log_10_ CCU/mL	0.62
AUC_24h_/MIC for 2 Log_10_ CCU/mL	1.33
AUC_24h_/MIC for 3 Log_10_ CCU/mL	1.49
Slope (N)	4.78

E_0_ is the change in Log_10_ CCU/mL after 24 h incubation in the control sample (absence of drug) compared with the initial inoculum. E_max_ is the difference in effect of the greatest amount of kill. EC_50_ is the AUC_24h_/MIC value producing a 50% reduction in bacterial counts from the initial inoculum. AUC_24h_/MIC is the 24 h area under concentration-time curve/minimum inhibitory concentration ratios. N is the Hill coefficient that describes the steepness of the AUC_24h_/MIC-effect curve.

## DISCUSSION

*M. gallisepticum* is one of the primary pathogens that cause respiratory diseases in poultry, and severely impacts the development of commercial poultry production. Drug resistance to *M. gallisepticum* has emerged in recent years, due to long-term improper use of antibacterial agents (Morrow et al., 2020). Therefore, there is an urgent need to further explore new treatments and control methods. Our previous study demonstrated that the preventive effects of baicalin against *M. gallisepticum*-induced inflammation and apoptosis in the lungs of chicken (Wu et al., 2019; Ishfaq et al., 2020). In the current study, we established an in vivo PK/PD profile of baicalin and evaluated the efficacy of baicalin against *M. gallisepticum* infection model.

Our choice for inoculation routes and the concentration of *M. gallisepticum* were based on previous studies. We compared four different *M. gallisepticum* infection models and found that the direct air sac injection is the faster and more effective method (Bao et al., 2020). The concentration of *M. gallisepticum* for pretest infection was the same as before, 5 × 10^9^ CCU/mL. Additionally, we considered the selection of the PDs calculation method. It is well understood that the isolation rate of *M. gallisepticum* is low, due to its high requirements on culture medium conditions and susceptibility to external contamination (Garcia et al., 1995). So, it may not be an effective method to evaluate the PD by isolation count. Studies have confirmed that it is more convenient and efficient to use real-time PCR methodology to detect *M. gallisepticum* quantitatively, and it can be used to evaluate the PD parameters (Xiao et al., 2015; Xiao et al., 2016). Thus, in this study, we choose to detect *M. gallisepticum* quantitation by qRT-PCR separately before and after administration, which may provide a more accurate estimation of the difference in PDs.

To obtain a satisfactory analytical method, chromatographic conditions including the composition, pH of mobile phase, and apparatus were all optimized after several trials and referred to some previous studies (Xu et al., 2020; Zhu et al., 2021). We compared the methods and found that the obtained baicalin target peak response value was higher when using 0.4% (v/v) phosphoric acid (A) and methanol (B) as the mobile phase method. It may be due to the weak acidity of baicalin. A certain amount of phosphoric acid can increase the resolution of baicalin and inhibit its dissociation. Meanwhile, we found that adding triethylamine in the mobile phase can prevent the target peak from tailing and improve the peak shape. In addition, the determination method by HPLC usually took too much time (>40 min), and was not sensitive enough. Taking into account the high protein binding rate of baicalin in vivo, we switched to UPLC for baicalin quantification. It is found that on the basis of the same mobile phase conditions, using UPLC has higher sensitivity and shorter retention time.

To the best of our knowledge, this was the first report about the PK/PD of baicalin in *M. gallisepticum*-infected chickens. In the present investigation, PK profiles of baicalin ranged from 200 mg/kg to 600 mg/kg has been described using baicalin concentration in the plasma and lung tissues in infected chickens. Baicalin was rapidly absorbed with the peak concentrations achieved at 0.75 h. The half-life was in the range of 10.93 to 17.80 h in plasma and in the range of 6.86 to 10.72 h in the lung, which was lower than the value of previously reported studies (Wei et al., 2017; Li et al., 2018). Hence, it indicated that the elimination rate of baicalin was significantly different among species. The PK profiles revealed that the average baicalin concentration in lung tissues was higher (1.21–1.73 times higher) than the corresponding concentration in plasma. It is consistent with the previously published article that higher concentrations of baicalin in lung tissues of rabbits and rats were noted compared to plasma (Zhu et al., 2013; Wei et al., 2016). The difference in the concentration of baicalin in the lung tissues may be due to transporters (Kalapos-Kovács et al., 2015). Baicalin can be transferred to the alveolar space as a substrate through a variety of transport proteins.

In this experiment, baicalin was rapidly absorbed in the body, the first absorption peak appeared at 0.75 h, and the second absorption peak appeared at about 8 h. Many studies have given multiple mechanisms to explain this bimodal phenomenon. The previous study showed after oral or intravenous injection of baicalin, it undergoes a glucuronidation reaction in rats, and the hepatoenteric circulation has a great influence on the absorption and metabolism of baicalin (Xing et al., 2005). Some researchers have also proposed that absorption sites and intestinal flora may also be responsible for the multipeak absorption of baicalin. Experiments have shown that the absorption of baicalin was segment dependent (Fong et al., 2012). In addition, the biotransformation and the mechanism of action of baicalin in vivo is closely related to the intestinal flora that produces metabolic enzymes (Noh et al., 2016; Wang et al., 2021a).

The parameters of baicalin (AUC_24h_) showed dose proportionality in the range of 200 to 600 mg/kg following administration allowed us to calculate the AUC_24h_ for other dosage administrations. We choose AUC_24h_/MIC as the PK/PD index of baicalin, since the data from the present multiple dosage studies confirmed the conclusion that baicalin is a concentration-dependent drug (Cheng et al., 2014). Our data showed a stronger correlation between the AUC_24h_/MIC and the in vivo antibacterial effects of baicalin against *M. gallisepticum* (R^2^ = 0.959) than C_max_/MIC (R^2^ = 0.898). The AUC_24h_/MIC ratios for *M. gallisepticum* (a reduction of 0 Log_10_ CCU/mL), a reduction of 2 Log_10_ CCU/mL, and a reduction of 3 Log_10_ CCU/mL were 0.62 h, 1.33 h, and 1.49 h, respectively. In addition, a PK population clearance of baicalin should be calculated and the MIC distribution of *M. gallisepticum* to baicalin should be evaluated. In the clinic, the parameters of this study can be used to formulate dosing schedules in combination with the different immune functions condition.

In conclusion, the present study successfully constructed a synchronous PK/PD model of baicalin in chickens infected with *M. gallisepticum*. The in vivo data suggested that baicalin concentration in lung tissues was higher than plasma in *M. gallisepticum*-infected chickens (1.21–1.73 times higher). Taken together, baicalin showed therapeutic potential against *M. gallisepticum* infection in chickens, and the values of AUC_24h_/MIC of baicalin against bacteriostatic, bactericidal, and eradication were 0.62, 1.33, and 1.49 h, respectively.
